# Stress Response of *Mesosutterella multiformis* Mediated by Nitrate Reduction

**DOI:** 10.3390/microorganisms8122003

**Published:** 2020-12-15

**Authors:** Nao Ikeyama, Moriya Ohkuma, Mitsuo Sakamoto

**Affiliations:** 1 Microbe Division/Japan Collection of Microorganisms, RIKEN BioResource Research Center, Tsukuba 305-0074, Ibaraki, Japan; nao.ikeyama@riken.jp (N.I.); mohkuma@riken.jp (M.O.); 2 PRIME, Japan Agency for Medical Research and Development (AMED), Tsukuba 305-0074, Ibaraki, Japan

**Keywords:** *Mesosutterella multiformis*, the family *Sutterellaceae*, nitrate reduction, nitrate, nitrite, filament formation, stress response

## Abstract

Bacterial stress responses are closely associated with the survival and colonization of anaerobes in the human gut. *Mesosutterella multiformis* JCM 32464^T^ is a novel member of the family *Sutterellaceae*, an asaccharolytic bacterium. We previously demonstrated energy generation via heme biosynthesis, which is coupled with nitrate reductase. Here, physiological and morphological changes in *M. multiformis* induced by exposure to nitrate were investigated. The ability of *M. multiformis* to reduce nitrate was determined using a colorimetric assay. A unique morphology was observed during nitrate reduction under anaerobic conditions. The association between nitrate concentration and cell size or cellular fatty acid composition was evaluated. Nitrate-induced responses of *M. multiformis* were compared to those of related species. An increase in cellular filamentation and the ratio of saturated: unsaturated fatty acids was mediated specifically by nitrate. This indicates a decrease in cell fluidity and low leakage. Furthermore, a similar response was not observed in other related species cultured in the presence of nitrate. Hence, the nitrate-induced stress response in new anaerobes such as *M. multiformis* was demonstrated. The response could also be involved in the conservation of menaquinones and the maximization of nitrate reduction.

## 1. Introduction

For a variety of microorganisms, it is important to survive in the human gut, a highly complex ecosystem. They may frequently encounter antibiotics or other potentially toxic components secreted by surrounding organisms in nutrient-deficient or energy-limited environments. Many reports have focused on using lactic acid bacteria as starter cultures for the production of fermented food and probiotics, or saccharolytic bacteria acting as foodborne pathogens and causing clinical infections [1]. Bacterial stress responses are diverse and are mediated by various factors (including pH, temperature, osmotic pressure, alcohol, starvation, and antibiotics) in unfavorable environments. This indicates that damage to cellular integrity, cell membrane composition, viability, as well as disruption of gene regulation leads to changes in their phenotypes [2]. With respect to morphological changes, sporulation influences the thickness of the cell wall and cell size. The stress response of Gram-negative bacteria is associated with the integrity of the cell envelope and bacterial toxicity [3]. Understanding the bacterial SOS response in a stress-inducing environment could be the key to revealing their physiological functions and the survival strategy adapted to colonize the human gut.

*Mesosutterella multiformis*, an asaccharolytic, obligately anaerobic, and Gram-negative bacterium, was isolated from the fecal samples of healthy Japanese individuals [4]. This species is a novel member of the family *Sutterellaceae*. Based on phylogenetic analyses, it is located between the genera *Parasutterella* and *Sutterella*. *Sutterellaceae* species are anaerobic or microaerophilic and their role in the human gut remains unclear. However, an association between *Sutterella* species and gastrointestinal disturbances in children with autism [5] or mild pro-inflammatory capacity in the human gastrointestinal tract [6] has been demonstrated. An association between *Parasutterella* species and irritable bowel syndrome or intestinal chronic inflammation has also been reported [7]. These species are present in abundance in the healthy human colon.

Recently, we demonstrated energy production in *M. multiformis* mediated by the proton motive force via the heme biosynthesis pathway [8]. In this pathway, the activity of nitrate reductase serves as the key component [9]. The nitrate metabolism of gut bacteria is important in elucidating their symbiotic properties [10].

In this study, we investigated the physiological and morphological changes in *M. multiformis* under stress conditions *in vitro* and compared such alterations with related species. To our knowledge, no reports have been published on the stress response of *Sutterellaceae* species isolated from human feces. Our findings reveal one of the stress responses of asaccharolytic bacteria such as *M. multiformis* that are difficult to cultivate owing to the unknown metabolic requirements for growth. This could complement the description of the metabolism of *M. multiformis* provided in our previous report.

## 2. Materials and Methods

### 2.1. Bacterial Strains and Growth Conditions

*M. multiformis* JCM 32464^T^, *Sutterella megalosphaeroides* JCM 32470^T^, *Sutterella parvirubra* JCM 14724^T^, *Sutterella stercoricanis* JCM 32441^T^, *Sutterella wadsworthensis* JCM 32440^T^, *Parasutterella excrementihominis* JCM 15078^T^, and *Parasutterella secunda* JCM 16078^T^ were obtained from the Japan Collection of Microorganisms (JCM), RIKEN BioResource Research Center, Tsukuba, Japan. All strains were maintained on Brucella blood agar supplemented with hemin and menadione (BB; JCM Medium No. 677) for 2–4 days at 37 °C under a H_2_/CO_2_/N_2_ (1:1:8, by vol.) gas mixture. As *M. multiformis* cannot grow in a liquid medium, TSA plates were used as a solid medium to facilitate growth. In addition, potassium nitrate (KNO_3_; nitrate) or sodium nitrite (NaNO_2_; nitrite) were added to the media as required.

### 2.2. Determination of Nitrite Concentrations

The strain was plated on TSA containing different concentrations of nitrate (0, 0.1, 0.5, 1, 10, and 50 mM) to examine nitrate reduction ability. Nitrite concentrations were determined using a colorimetric assay [11]. The reagents comprised 1 part 0.4% sulfanilic acid plus 30% of acetic acid mixed with H_2_O and 1 part 0.6% *N*,*N*-dimethyl-1-naphthylamine plus 30% of acetic acid mixed with H_2_O. The bacterial colonies grown on the TSA were collected with a cotton swab after 3, 6, and 10 days of incubation and suspended in 1 mL of distilled water. The optical density of these suspensions was measured at 660 nm (OD_660_) using an Ultrospec 2100 *pro* spectrophotometer (Amersham Biosciences, Piscataway, NJ, USA) and adjusted to approximately 0.1. A total of 1 mL of cell suspension was mixed with 20 µL of each reagent and incubated for 1 h at room temperature. The absorbance was measured at 540 nm using a spectrophotometer, and distilled water with reagents was used as a blank. Nitrite concentrations were calculated from a calibration curve that was constructed using several known concentrations of the nitrite solution.

### 2.3. Evaluation of Cell Morphology

To observe cellular shape during nitrate respiration, *M. multiformis* JCM 32464^T^ was grown on TSA supplemented with additional nitrate or nitrite as required and on a BB plate for Gram staining. The cells were observed using light microscopy (BIOPHOT, Nikon, Tokyo, Japan). The open-software platform ImageJ (https://imagej.nih.gov/ij/) was used to measure the cell length by direct counts of more than 300 bacterial cells and to compare the dispersion of filamentous cells for each treatment. Subsequently, the morphology of cells was observed after six days of culture on TSA supplemented with 10 mM nitrate using scanning electron microscopy (SEM; JEM-6340F; JEOL, Tokyo, Japan) and transmission electron microscopy (TEM; JEM-1400plus; JEOL, Tokyo, Japan). Sample preparation for SEM and TEM has been described previously [12].

### 2.4. Effect of Nitrate on the Composition of Cellular Fatty Acids

We next determined the differences in the composition of cellular fatty acids and morphological changes. Fatty acid methyl esters (FAMEs) were obtained from approximately 40 mg of wet cells grown on TSA without nitrate or with 1, 10, and 50 mM of nitrate at 37 °C for 6–8 days by saponification, methylation, and extraction with minor modifications [13] using the method described by Millar [14]. The composition of cellular fatty acid was determined using version 6.2B of the Sherlock Microbial Identification System (MIDI) and version 3.80 of the BHIBLA database.

### 2.5. Evaluation of the Ability of Other Sutterellaceae Species to Form Filaments

Other reference strains were evaluated for their ability to form filaments on TSA in the presence of nitrate or nitrite. Aliquots of the culture were observed after four days of incubation using light microscopy to determine whether filaments were formed. Cultivation of all strains was performed under the same conditions.

### 2.6. Genome Analysis

The genome data of *S. megalosphaeroides* JCM 32470^T^, *S. wadsworthensis* 2_1_59BFAA, *S. parvirubra* YIT 11816^T^, and *P. excrementihominis* YIT 11859^T^ were obtained from the NCBI database. *S. stercoricanis* and *P. secunda* were excluded from several comparisons owing to the unavailability of genome data in the database. The genes were analyzed by using the Kyoto Encyclopedia of Genes and Genomes (KEGG; release 63.0) [15], Rapid Annotations using Subsystem Technology (RAST; v. 2.0) [16], and the DDBJ Fast Annotation and Submission Tool (DFAST; v. 1.0.2) [17]. Default settings were used.

### 2.7. Statistical Analyses

Statistical differences and significance were assessed using the ANOVA test. Student’s *t*-test and post hoc Bonferroni test were used to analyze paired data, and the *p*-values were obtained. To determined significances in nitrite concentration among each treatment, Tukey’s HSD (honestly significant difference) test was performed.

## 3. Results

### 3.1. Effect of Nitrate Concentration on Ability of Nitrate Reduction

**Figure 1:**
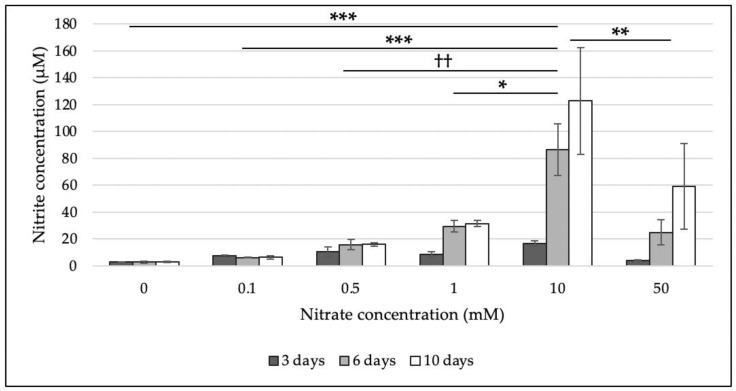
Determination of nitrite concentration using a colorimetric assay. The bacterial cells grown on TSA supplemented with different nitrate concentrations (0, 0.1, 0.5, 1, 10, and 50 mM) after 3, 6, and 10 days of incubation were used. Nitrite concentrations (µM) were determined by measuring the absorption at 540 nm. Experiments were performed in triplicates. Error bars represent the standard deviation between biological replicates. The statistical significances (*p* < 0.05) among different concentrations of nitrate that were determined by Turkey’s HSD test are shown. ***, significant differences in all incubation stages; **, significant differences in 3 and 6 days; ††, significant differences in 6 and 10 days; *, significant difference in 6 days.

The ability of *M. multiformis* JCM 32464^T^ to reduce nitrate was evaluated using a colorimetric assay. *M. multiformis* JCM 32464^T^ was able to reduce nitrate to nitrite. The results are in accordance with the prediction of the presence of a nitrate reductase gene based on genome analysis [8]. *M. multiformis* JCM 32464^T^ was incubated on TSA with different nitrate concentrations (0, 0.1, 0.5, 1, 10, and 50 mM). The detection of a significant nitrite concentration was observed with 10 mM of nitrate (Figure 1), and 122.89 µM nitrite concentration was produced after 10 days. In contrast, cultures with 50 mM nitrate resulted in the production of up to 59.18 µM nitrite, indicating the inhibition of nitrate reduction.

### 3.2. Effect of Nitrate on Cell Morphology of M. multiformis

**Figure 2:**
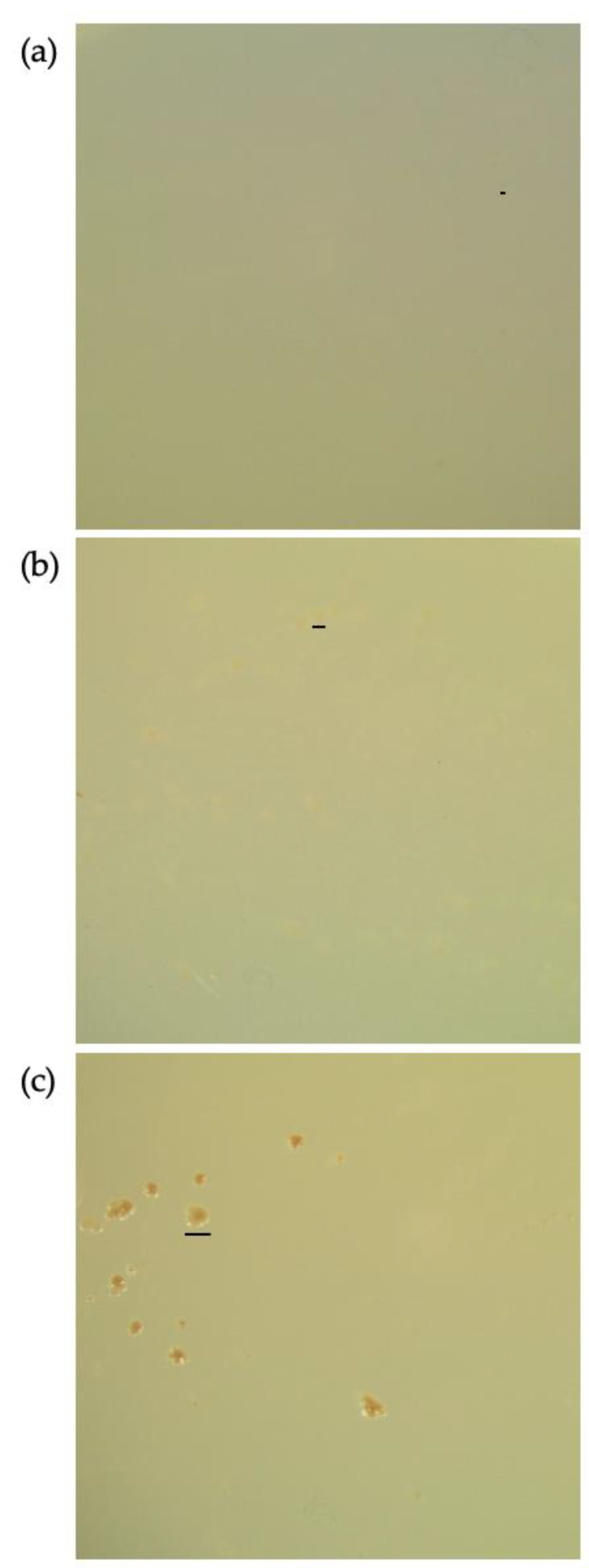
Pigment production by *Mesosutterella multiformis* in the presence of nitrate. The strain was cultured on TSA for 15 days at 37 °C under a H_2_/CO_2_/N_2_ (1:1:8, by vol.) gas mixture. (**a**) Culture in the absence of nitrate, (**b**) culture in the presence of 1 mM of nitrate, and (**c**) culture in the presence of 50 mM of nitrate. Bars, 0.2 mm (**a**), 0.5 mm (**b**), or 1 mm (**c**).

The colonies of *M. multiformis* JCM 32464^T^ cultured on TSA in the presence of nitrate differed from those cultured on TSA without nitrate. Although the total number of colonies was decreased, the formation of colony variants increased. The colonies were larger (0.1–1 mm) than the regular size (0.1–0.2 mm). After long-term incubation with nitrate, the colonies produced a small amount of brown pigment (Figure 2c).

**Figure 3:**
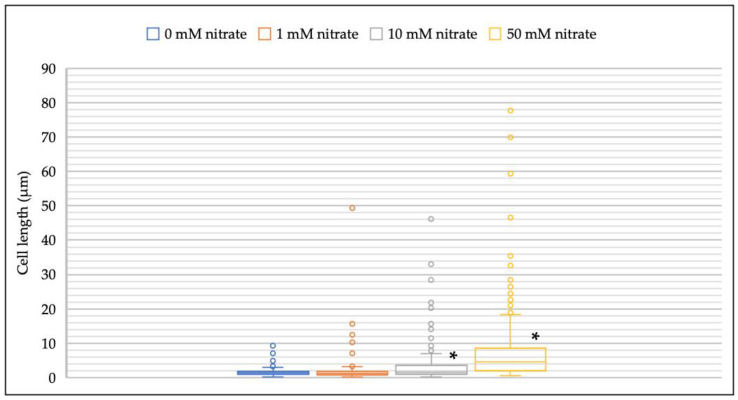
Distribution of cell lengths of *M. multiformis* at different nitrate concentrations. The strain was grown on TSA supplemented with 0–50 mM of nitrate. More than 300 cells of *M. multiformis* were selected and analyzed directly using ImageJ. * indicates significant differences (*p* < 0.05) compared with control (0 mM nitrate) by Student’s *t*-test and post hoc Bonferroni test.

The addition of nitrate significantly increased the elongation of *M. multiformis* JCM 32464^T^ cells compared to the bacterial colonies cultured on TSA in the absence of nitrate (Figure 3). Compared to 1.7–2.1 µm of regular cell length, the length of the filamentous cells was approximately 80 µm. Cell filamentation was frequently observed with 10 mM of nitrate concentration, and the range of cell lengths was higher at 50 mM of nitrate concentrations compared to that observed at all other concentrations. Filamentous cells were not observed when cultured on BB and TSA in the absence of nitrate with no change in cellular morphology.

**Figure 4:**
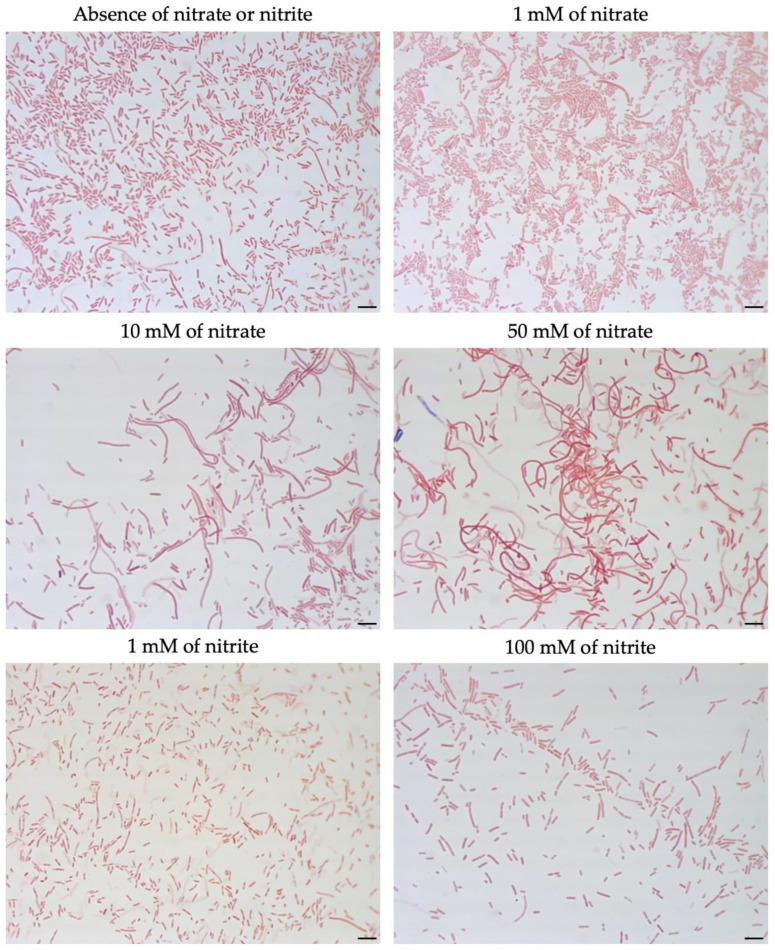
Morphology of *M. multiformis* cells grown on TSA in the presence of different concentrations of nitrate and nitrite detected by Gram-staining. Concentrations of 1, 10, and 50 mM of nitrate and 1 and 100 mM of nitrite were added on TSA. The strain was grown on TSA for 4 days at 37 °C under anaerobic conditions. Bars, 10 µm.

To evaluate whether nitrite plays a role in filament formation, nitrite was added to the TSA. The addition of 100 mM nitrite to TSA did not have a significant effect on the morphology of *M. multiformis* JCM 32464^T^ (Figure 4). Notably, when filamentous cells of *M. multiformis* JCM 32464^T^ were transferred to fresh BB plates, the cell size was recovered, and a size similar to regular cells was observed.

**Figure 5:**
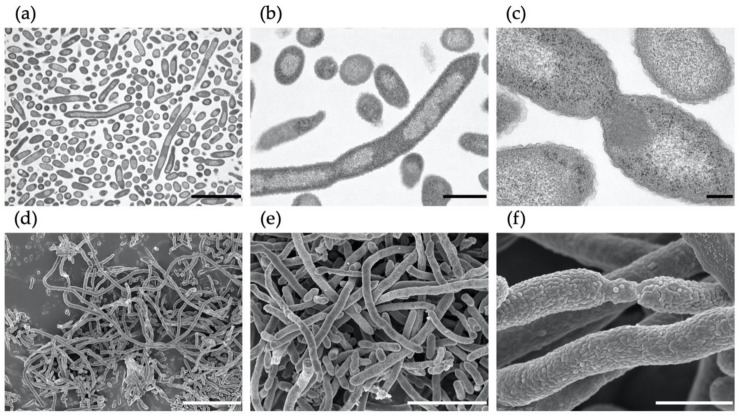
Electron micrographs of *M. multiformis* cells cultured with 10 mM nitrate after 6 days of incubation. Images were obtained using transmission electron microscopy (**a**–**c**) and scanning electron microscopy (**d**–**f**). Bars, 5 µm (**a**,**e**), 1 µm (**b**,**f**), 200 nm (**c**), or 10 µm (**d**).

Scanning and transmission electron micrographs are shown in Figure 5. Intracellular shading was observed by TEM images. Moreover, entangled masses of cells were also observed sporadically. These results suggest that these morphological changes are caused by nitrate.

### 3.3. Effect of Nitrate-Induced Filamentation on the Composition of Cellular Fatty Acids

**Figure 6:**
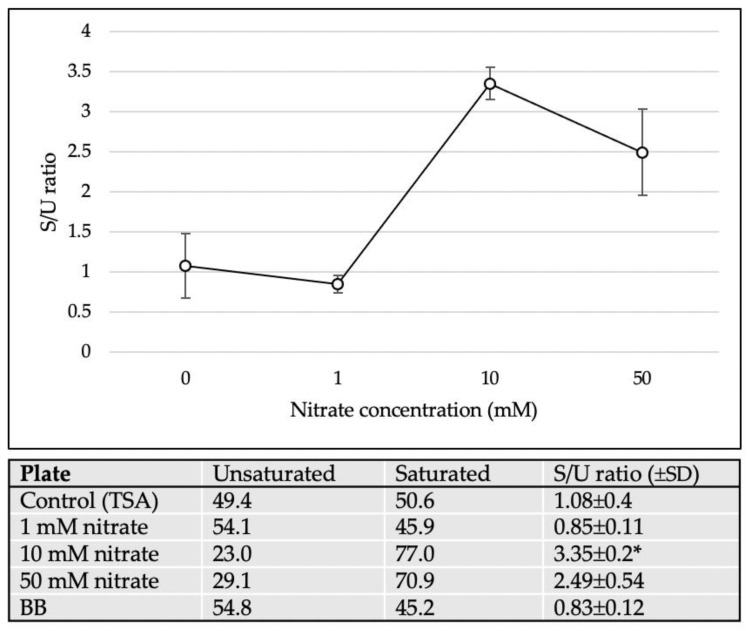
The S/U ratio of *M. multiformis* cultured with different nitrate concentrations. The saturated fatty acids, namely, C_9:0_, C_12:0_, C_14:0_, C_16:0_, C_18:0_, C_16:0_ ALDE, C_16:0_ DMA and summed feature 5 (C_15:0_ DMA and/or unknown 17.834); the unsaturated fatty acids, namely, C_16:1_*ω*7c and summed feature 10 (C_18:1_c11/t9/t6 and/or unknown 17.834). All values and error bars represent the mean ± standard deviation of three experiments. * indicates significant differences (*p* < 0.05) compared with control (0 mM nitrate) by Student’s *t*-test and post hoc Bonferroni test.

The differences in cellular fatty acid composition are shown in Table 1. C_16:0_, summed feature 10 (C_18:1_c11/t9/t6 and/or unknown 17.834), C_14:0_, and C_16:1_*ω*7c were the major cellular fatty acids (>10%) of *M. multiformis* JCM 32464^T^ cultured on TSA in the presence of nitrate. Interestingly, with 10 mM of nitrate, the amount of C_14:0_ observed was higher than that observed with other treatments (2.3 to 23.0% compared to the control). As expected, this pattern was different from the profiles of *M. multiformis* JCM 32464^T^ cultured without nitrate. Instead of the increase in the amount of C_16:0_ (32.8% to 48.5%), the amount of summed feature 10 decreased from 44.1% to 18.1% upon the addition of 50 mM of nitrate. Furthermore, the ratio of saturated fatty acid to unsaturated fatty acid (S/U ratio) significantly increased with the addition of 10 or 50 mM nitrate (Figure 6). These results indicated that the fluidity of cell membranes was decreased [18]. However, a lower S/U ratio was observed when 1 mM of nitrate was used as a relative control.

### 3.4. Effect of Nitrate or Nitrite on Other Sutterellaceae Species

**Figure 7:**
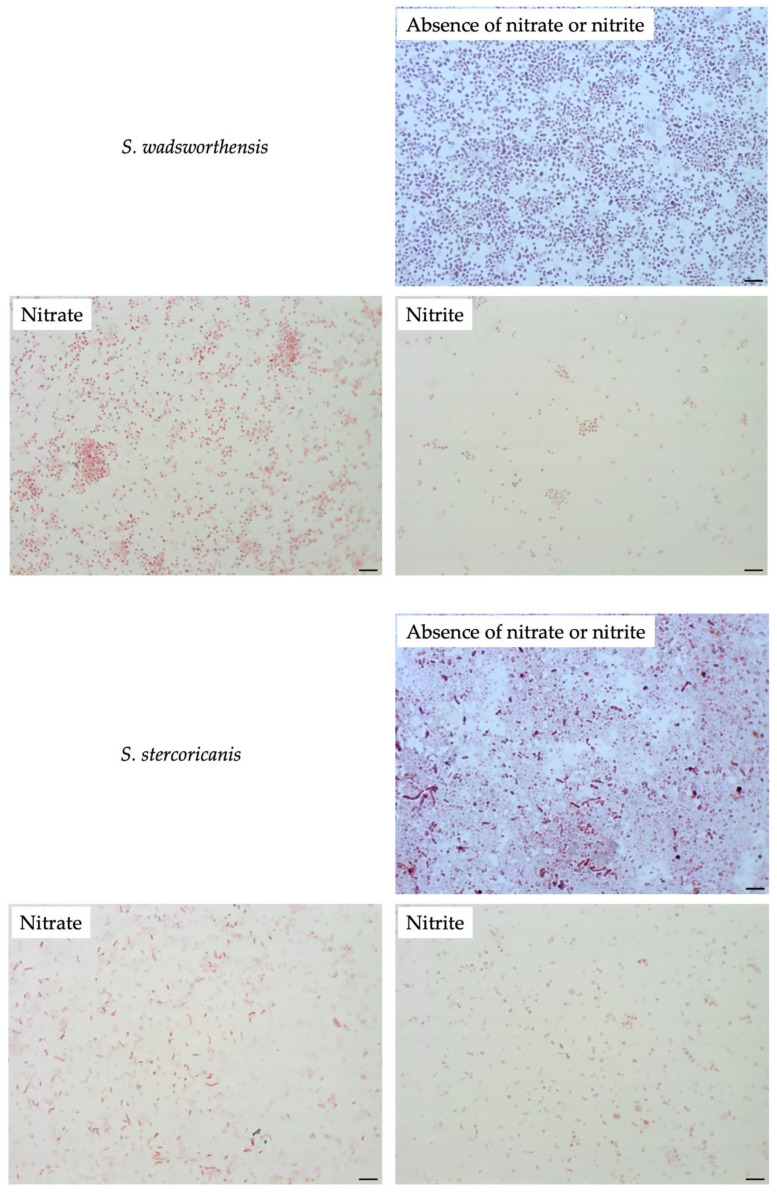

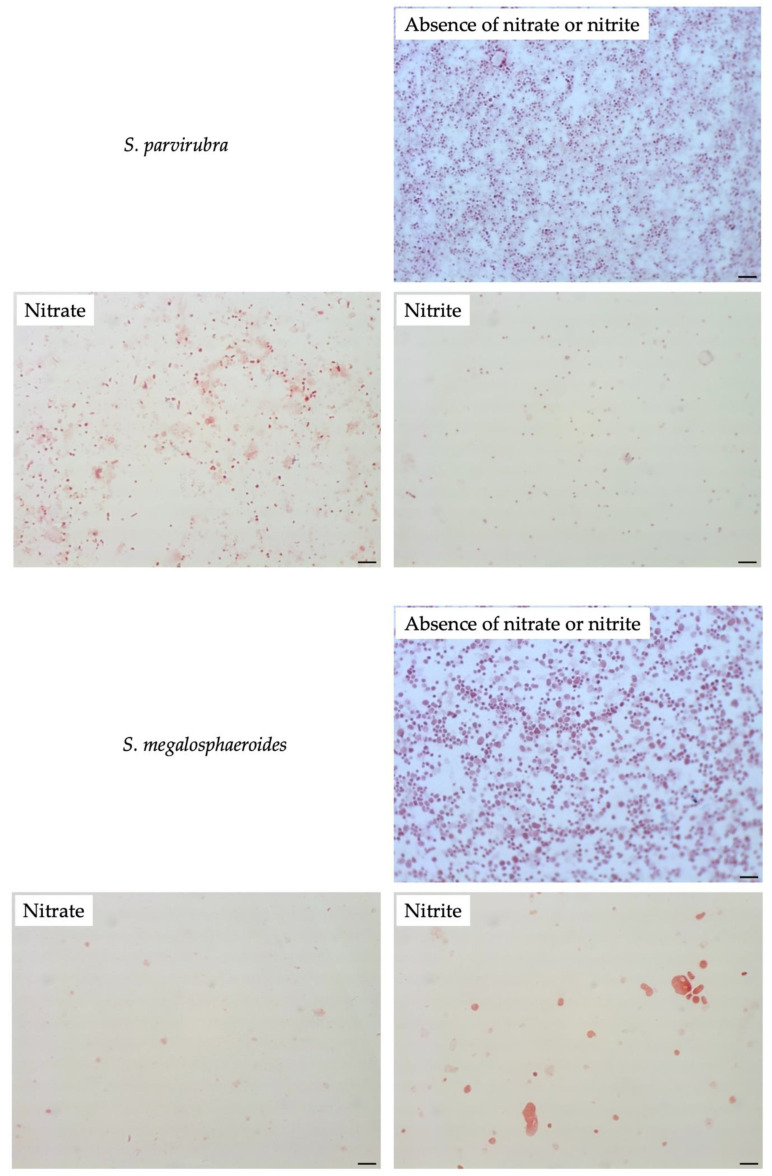

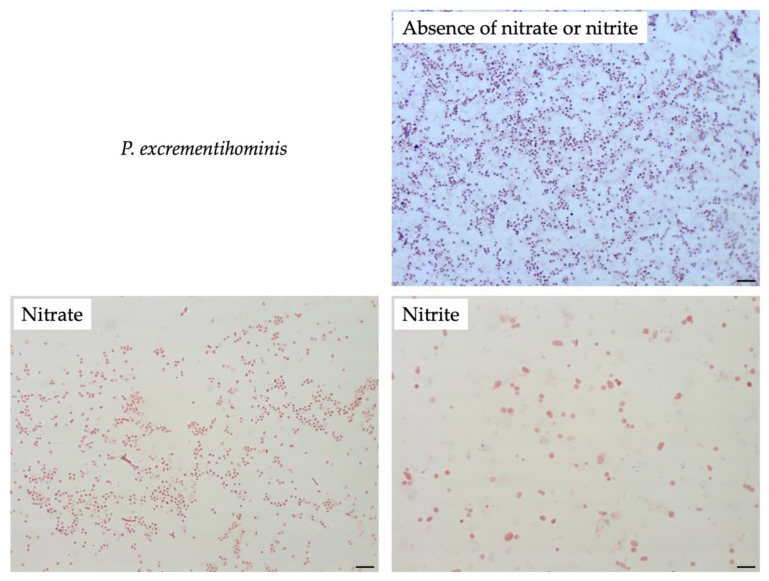
Cellular morphology of reference strains cultured in the presence of nitrate or nitrite observed by Gram-staining after four days of incubation at 37 °C under anaerobic conditions. None represent TSA only; Nitrate represents supplementation with 50 mM of nitrate; Nitrite represents supplementation with 1 mM of nitrite. Bars, 10 µm.

To compare filamentation ability, other *Sutterellaceae* species were exposed to nitrate or nitrite. No similar elongation was observed in the presence of 50 mM nitrate with other reference strains. However, in the presence of 1 mM of nitrite, *S. megalosphaeroides* JCM 32470^T^ and *P. excrementihominis* JCM 15078^T^ cells demonstrated swelling and burst-like morphology (Figure 7). The swelling cells of *P. excrementihominis* JCM 15078^T^ showed 2.0–2.6 µm (nitrite) from 0.8 to 1 µm (absence of nitrate or nitrite) and *S. megalosphaeroides* JCM 32470^T^ with nitrite showed 3.0–8.7 µm from 1 to 2 µm (absence of nitrate or nitrite).

### 3.5. Differences of Genes in Sutterellaceae Species

The draft genomes of *M. multiformis* and *P. excrementihominis* demonstrated genes involved in heme biosynthesis (Table 2), such as nitrate reductase, *hemG*, ATPase, and menaquinones that were coupled to electron transport chains for energy generation [9,19]. *M. multiformis* and *P. excrementihominis* showed longer chain menaquinones (MK-6 and MMK-6) [4]. In addition, *S. megalosphaeroides*, *S. wadsworthensis*, and *S. parvirubra* were predicted to possess a heme biosynthesis pathway, but did not possess genes encoding nitrate reductase. The genes encoding nitrite reductase were located in the genome of *M. multiformis*. This strain has the ability to convert nitrite to ammonia in the nitrogen cycle, but not the ability to produce nitric oxide.

## 4. Discussion

*M. multiformis* JCM 32464^T^ showed a unique cellular morphology influenced by nitrate that has not been explored before.

Although *M. multiformis* formed small pinpoint colonies on common blood agar plates, cell growth was arrested in the liquid medium. Related *Sutterella* spp. grow poorly and show no visible turbidity in broth [24]. These findings indicate a lack of growth factors in the liquid medium to facilitate the growth of *Sutterella* spp.

A recent study examined the ability of *Sutterella* spp. to adhere to intestinal epithelial cells [6]; however, the association between their adherence and growth in the gut remains unclear.

We previously suggested that *M. multiformis* generated a trace amount of ATP via heme biosynthesis [8]. Menaquinone is utilized as an electron donor for the reduction of nitrate to nitrite mediated by nitrate reductase, which is the key enzyme in this pathway [9]. However, in this study, their growth was not enhanced by additional nitrate, indicating that nitrate was not adapted to growth stimulation.

The addition of nitrate induced filamentation in *M. multiformis* JCM 32464^T^ cells. This unique morphology was adapted to cope with nitrate stress. Morphological changes such as filament formation are a type of stress response observed under specific conditions. For instance, changes are observed in *Salmonella enterica* with pelargonic acid [25], *Pseudomonas aeruginosa* during anaerobic respiration [26], *Corynebacterium glutamicum* with nitrate [27], and *Bacillus subtilis* during anaerobic nitrate respiratory [28]. The changes might be attributed to defects in cell division, DNA damage, or the induction of SOS gene expression. A higher concentration of nitrate in the medium demonstrated a lower nitrite production and increased cell length, suggesting that the elongation of *M. multiformis* was a stress response toward nitrate. Once the filamentous cells were cultured on TSA without nitrate, cellular morphology was almost recovered, and morphology similar to regular cells was observed. This result demonstrated that the filamentous cells could be fragmented again and their biological activity could be recovered.

SEM images showed that the elongation of *M. multiformis* was accompanied by abnormal cell division, with intracellular light and shade. Clump formation was occasionally observed in the presence of nitrate (Figure 5) similar to *S. wadsworthensis* [29], which can be attributed to both the presence of excessive nitrate and microaerobic conditions.

These morphological changes affected the structure or surface of bacterial cells. One of the stress responses altered the composition of cellular fatty acids. The level of unsaturated fatty acids is correlated with the fluidity of the cell membrane [30]. In a previous study, the association between cell membrane composition and synthesis of menaquinones in *Escherichia coli* was investigated [18]. Liu et al. showed that an increase in the S/U ratio promoted the expression of intracellular menaquinones, and unsaturated fatty acids stimulated the secretion of extracellular menaquinones. The shorter lipids (C_16:0_, C_14:0_) and the S/U ratio increased upon the addition of 10 or 50 mM nitrate (Table 1, Figure 6). This indicated that the membrane of filamentous cells demonstrated low leakage and conserved menaquinones. This result is consistent with nitrite production and the S/U ratio as inferred from the role of menaquinones involved in nitrate respiration [31]. Consequently, it might be one of the strategies employed for maximizing menaquinone utilization for nitrate reduction.

Normally, BB plates are used for the cultivation of *M. multiformis*. The cellular fatty acid composition (S/U ratio) on BB plates was similar to that observed on TSA in the presence of 1 mM nitrate compared to the control [4]. This result indicated that an appropriate amount of nitrate supported the growth and cellular stability of *M. multiformis*.

These stress mechanisms causing changes in cell surface and membrane composition may be associated with adhesion to intestinal cells [32,33]. As mentioned above, the adherence of *Sutterella* spp. is closely related to human intestinal health.

The genome of all related species was predicted to contain *hemG* involved in the heme biosynthesis pathway. Only *M. multiformis* and *P. excrementihominis* possess the gene *narGHJI* encoding nitrate reductase, similar to the longer-chain menaquinones (MK-6 and MMK-6). However, some discrepancies were observed in the nitrite tolerance of *P. excrementihominis* compared to *M. multiformis*. Cells of *M. multiformis* did not demonstrate any changes when cultured on the TSA containing 100 mM of nitrite, whereas *S. megalosphaeroides* and *P. excrementihominis* cells showed burst morphology in the presence of 1 mM of nitrite (Figure 7). Moreover, *P. secunda* did not form colonies with nitrite. Nitrite is known to have antimicrobial properties, and the ability to exert toxic effects might be a possible mechanism [34]. Nitrite is added to inhibit the growth of bacterial pathogens in processed meat. Nitrate is also included in many edible plants; hence, the dietary intake of nitrate can be a precursor to nitrite production in the gut.

Only *M. multiformis* showed elongated cell morphology influenced by nitrate among all related species. These morphological changes were specifically observed in the presence of nitrate; however, filamentation might be induced by certain environmental factors in the human gut [35,36,37]. The family of *Sutterellaceae* was highly detected in the biopsy of patients of IBD and autism by metagenomic analysis [38]. However, we have isolated several strains within the family *Sutterellaceae* including *S. parvirubra*, *S. wadsworthensis*, *S. megalosphaeroides*, and *M. multiformis* from healthy human feces. Thus, this species was commonly abundant in the human gut regardless of the presence or absence of disease. This suggested that this physiological and morphological alteration induced by various intestinal environments such as high nitrate concentration correlated their abundance and biological activity in the human gut. Host–microbe interactions with filamentous cells should be examined in further studies.

Considering the difficulty to obtain a sufficient amount of RNAs because of their poor growth of *M. multiformis*, gene expression could not be confirmed by transcriptome analysis, as the current growth condition. Further studies are required to determine the growth factors that are needed to prepare enough bacterial samples for more multidimensional experiments.

These findings revealed that excessive nitrate could be a factor involved in inducing the stress response in *M. multiformis*, leading to filamentation and the inhibition of enzyme activity. The cellular fatty acid compositions were significantly altered during cell elongation as a stress response, suggesting strategies for maximizing nitrate respiration and menaquinone conservation to compete with the excessive nitrate.

We believe that our findings have provided substantial insights into *M. multiformis* and will lead to a more comprehensive understanding of this uncharted organism.

## Figures and Tables

**Table 1:** Differential compositions of cellular fatty acids of *M. multiformis* cultured with or without nitrate.

Fatty Acids	0 mM Nitrate	1 mM Nitrate	10 mM Nitrate	50 mM Nitrate
Saturated:				
C_9:0_	1.4	-	-	3.2
C_12:0_	6.0	3.1	9.8 ± 8.1	3.9 ± 1.7
C_14:0_	2.3	3.1	23.0 ± 2.3 *	10.7 ± 2.5 *
C_16:0_	**32.8 ± 5.1**	**39.8 ± 7.2**	50.8 ± 4.2 *	48.5 ± 1.3 *
C_18:0_	9.4 ± 0.5	6.1	-*	5.8 ± 2.1
C_16:0_ ALDE	2.1	-	-	-
C_16:0_ DMA	3.8	-	-	-
Summed Feature 5 * (C_15:0_ DMA and/or unknown 17.834)	-	-	-	2.9
Unsaturated:				
C_16:1_*ω*7c	5.3 ± 0.6	6.6	**11.6 ± 0.8**	**11.0 ± 0.4**
Summed Feature 10 * (C_18:1_c11/t9/t6 and/or unknown 17.834)	**44.1 ± 9.0**	**49.7 ± 2.8**	11.5 ± 0.7 *	18.1 ± 3.9 *

*M. multiformis* JCM 32464^T^ was grown on TSA with 0, 1, 10, and 50 mM nitrate. −, not detected. ALDE, aldehyde; DMA, dimethyl acetal. Each value represents the mean ± standard deviation of three experiments. Major components (>10%) are highlighted in bold. * Summed features represent groups of two or three fatty acids that could not be separated using the MIDI system. * indicates significant differences (*p* < 0.05) compared with control (0 mM nitrate) by Student’s *t*-test and post hoc Bonferroni test.

**Table 2:** Differential characteristics of *M. multiformis* and related taxa.

Isolation Source		Nitrate Reductase	Nitrite Reductase	*hemG*	Major Quinones	Cell Mutation		References
						Nitrate	Nitrite	
*Mesosutterella multiformis*	Human feces	+	+	+	MK-6, MMK-6	+	–	[4]
*Sutterella megalosphaeroides*	Human feces	–	–	+	MK-5, MMK-5	–	+	
*Sutterella wadsworthensis*	Gastrointestinal tract infection	–	+	+	MK-5, MMK-5	–	–	[20]
*Sutterella stercoricanis*	Dog feces	NA	NA	NA	MK-5, MMK-5	–	–	[21]
*Sutterella parvirubra*	Human feces	–	–	+	MMK-5	–	–	[22]
*Parasutterella excrementihominis*	Human feces	+	–	+	MK-6, MMK-6	–	+	[23]
*Parasutterella secunda*	Human feces	NA	NA	NA	MK-5, MMK-5	–	NA	[24]

+, presence (or positive); −, absence (or negative); NA, not available.
